# An objective nodal staging system for breast cancer patients undergoing neoadjuvant systemic treatment

**DOI:** 10.1186/s12885-017-3380-8

**Published:** 2017-05-31

**Authors:** Tae-Kyung Yoo, Jung Min Chang, Hee-Chul Shin, Wonshik Han, Dong-Young Noh, Hyeong-Gon Moon

**Affiliations:** 1 Department of Surgery, Seoul National University College of Medicine, 03080, 101 Daehak-ro, Jongno-gu, Seoul, Republic of Korea; 2 Laboratory of Breast Cancer Biology, Cancer Research Institute, Seoul National University College of Medicine, 103 Daehak-ro, Jongno-gu, Seoul, Republic of Korea; 3 Department of Radiology, Seoul National University College of Medicine, 101 Daehak-ro, Jongno-gu, Seoul, Republic of Korea; 4 Department of Surgery, Chung-Ang University College of Medicine, 84 Heukseouk-ro, Dongjak-gu, Seoul, Republic of Korea; 5 Present address: Department of Surgery, Seoul St. Mary’s Hospital, College of Medicine, The Catholic University of Korea, 222 Banpo-daero, Seocho-gu, Seoul, Republic of Korea

**Keywords:** Breast cancer, Neoadjuvant systemic therapy, Nodal staging, Chest CT

## Abstract

**Background:**

In this study, we aimed to develop an objective staging system to determine the degree of nodal metastasis in breast cancer patients undergoing neoadjuvant systemic treatment (NST).

**Methods:**

We reviewed the pretreatment computed tomography (CT) images of 392 breast cancer patients who received NST. The association between the patterns of the enlarged regional lymph nodes and treatment outcome was analyzed.

**Results:**

In the development cohort of 260 patients, 88 (33.8%) patients experienced tumor recurrence and had a significantly higher number of enlarged lymph nodes on the pretreatment CT compared to patients with no recurrence. When patients were classified according to the numbers and locations of enlarged lymph nodes on pretreatment CT, the number of lymph nodes larger than 1 cm was most significantly associated with tumor recurrence. The accuracy of the CT-based nodal staging system was validated in an independent cohort of 132 patients. The presence of the enlarged supraclavicular nodes was associated with worse outcome, but the effect seemed to originate from the accompanied extensive axillary nodal burden. The prognostic effect of the objectively measured axillary nodal metastasis was more pronounced in hormone receptor-negative tumors.

**Conclusions:**

We have developed and validated an objective method of nodal staging in breast cancer patients who undergo NST based on the number of enlarged axillary lymph nodes. Our system can improve the current subjective approach, which uses physical examination alone.

**Electronic supplementary material:**

The online version of this article (doi:10.1186/s12885-017-3380-8) contains supplementary material, which is available to authorized users.

## Background

Neoadjuvant systemic therapy (NST) is increasingly used for the treatment of operable breast cancer in patients [1]. NST has been shown to increase the rate of breast conservation without compromising survival [2, 3]. The increased use of NST has given rise to some controversial issues such as the optimal method of determining the residual extent of tumor and the use of sentinel node biopsy after NST [4–6]. In this study, we raise another clinically important issue for breast cancer patients who receive NST: the issue of initial axillary staging. The decision for post-NST axillary management and adjuvant radiation therapy often relies on the initial axillary nodal status. As more patients with early breast cancer receive NST, the importance of accurate initial axillary nodal staging is increasing.

In breast cancer patients who undergo primary surgery, the number of metastatic lymph nodes is a major prognostic factor, and the risk of recurrence is proportional to the degree of disease burden in the axillary lymph nodes [7, 8]. In contrast, it is often difficult to obtain an accurate estimation of the extent of nodal involvement in patients who undergo NST. The current TNM staging system recommends physical examination to determine the N stage in patients receiving NST, based on the presence of the fixed nature or locations of the palpable nodes [9]. Unfortunately, studies have reported limited accuracy of physical examination in determining clinical N stage in patients with breast cancer, with a sensitivity around 30% [10–13]. Furthermore, clinical N staging with physical examination is a highly subjective method, and the accuracy may vary among surgeons.

In this study, we aimed to develop an objective staging system based on initial computed tomography (CT) images that can provide improved prognostic information for patients who receive NST.

## Methods

The medical records of patients who underwent NST and surgery for invasive breast cancer at Seoul National University Hospital (SNUH) between Jan 2006 and Dec 2011 were reviewed retrospectively. In our institution, patients who undergo neoadjuvant systemic therapy often received chest CT to rule out visceral metastasis and to assess the degree of nodal enlargement prior to the administration of the systemic treatment. For the present study, we excluded the patients who underwent palliative surgery or had a history of breast cancer. Also, patients who did not have chest CT images at diagnosis or who had poor-quality images were excluded. Patients were divided into two cohorts, in the ratio of two to one, for development and validation of a CT-based nodal staging system (development cohort and validation cohort, respectively). Patients’ data including clinicopathologic, treatment-related, and survival information were obtained from SNUH Breast Cancer Center database, which is a prospectively maintained web-based database [14]. In all individuals, CT scanning was performed at end-inspiration following hyperventilation. CT imaging was performed using the following scanners: Genesis Hispeed and LightSpeed Ultra; GE Healthcare, Milwaukee, WI, USA; Somatom Plus-4 and Sensation-16; Siemens Medical Systems, Erlangen, Germany; Brilliance-64; Phillips Medical Systems, the Netherlands. Two board-certified radiologists (mean 13 years of experience) who were blind to the clinical staging and treatment outcome information evaluated the CT scans independently. On the CT scans, lymph node status was evaluated based on the size and location (axillary levels I, II, and III, supraclavicular lymph node (SCN) or internal mammary lymph node (IMN)). At each location, the number of lymph nodes larger than 1 cm and larger than 2 cm was separately recorded. Discordant data from two radiologists were reevaluated by the same two radiologists to reach a consensus, and the final integrated results were recorded.

All patients were treated with anthracycline- and/or taxane-based chemotherapy regimens. Among the patients who had a human epidermal growth factor receptor 2 (HER2)-amplified tumor, 15% (17/113) received a HER2-related targeted therapy preoperatively. All patients received postoperative hormonal or radiation therapy, if indicated. Postoperative follow-up with the clinic was done at least every 6 months and included a routine physical examination, laboratory testing, breast ultrasonography, mammography and chest X-ray. Additional examinations were done at the physician’s discretion.

In this study, we used two additional groups of patients to assess the negative predictive value of CT evaluation and to estimate the hormone receptor (HRc)-dependent prognostic impact of nodal status. To assess the negative predictive value of the axillary nodal status as evaluated by chest CT, the medical records of breast cancer patients who received primary surgery as their initial treatment between January 2014 and December 2014 were reviewed for CT findings and pathological N staging (supplementary cohort A). To demonstrate the HRc-dependent prognostic effect, we selected consecutive patients who received primary surgery between July 2005 and June 2008 in whom adequate survival information was available (supplementary cohort B).

Univariate analysis, using the Pearson chi-square test, was performed to compare clinicopathologic features of the development and validation cohorts. Univariate survival analysis for development and validation of the CT-based nodal staging system was performed using Kaplan-Meier survival analysis and log-rank tests. Multivariate survival analysis was conducted using a Cox proportional hazards regression model. Prognostic factors with statistical significance in the univariate analysis were entered in the Cox regression model. Disease-free survival (DFS) was defined as the time from start of neoadjuvant systemic therapy to the date of breast cancer recurrence, death from any cause or final outpatient clinic visit. Breast cancer recurrence was defined as locoregional recurrence or distant metastasis, and contralateral breast recurrences were excluded.

## Results

### Patient characteristics and the prevalence of CT-detected lymph node enlargement

We reviewed the data of 536 breast cancer patients who received NST between January 2006 and December 2011. The median follow-up period was 63 months (range, 2–118 months). Among them 68 (12.7%) patients had metastatic disease at diagnosis, 1 (0.2%) patient had palliative surgery due to chest wall invasion and 2 (0.4%) patients had a history of breast cancer and were excluded. Also, 49 (9.1%) patients had no pre-NST chest CT scan, and 24 (4.5) patients had poor-quality images. After exclusion, a total of 392 patients were included in this study. The clinical and pathologic characteristics of the included patients are shown in Table 1. Clinically, 282 patients (71.9%) had stage III breast cancer.

**Table 1:** Clinical characteristics of the patients

		Development n (%)	Validation n (%)	*p*
		(*n* = 260)	(*n* = 132)	
Age (median, range)		46 (24–78)	46 (27–72)	
Breast Surgery	Breast conserving surgery	120 (46.2)	63 (47.7)	0.768
	Total mastectomy	140 (53.8)	69 (52.3)	
Axilla Surgery	Sentinel lymph node biopsy^a^	13 (5.0)	8 (6.2)	0.634
	Axillary lymph node dissection	247 (95.0)	122 (93.8)	
Clinical T stage	T1	3 (1.2)	4 (3.0)	0.061
	T2	126 (48.5)	54 (40.9)	
	T3	86 (33.1)	58 (43.9)	
	T4	45 (17.3)	16 (12.1)	
AJCC Stage	II	75 (28.8)	35 (26.5)	0.627
	III	185 (71.2)	97 (73.5)	
Histology	Ductal	237 (91.2)	121 (91.7)	0.528
	Lobular	6 (2.3)	5 (3.8)	
	Mixed/other	17 (6.5)	6 (4.5)	
Grade	Low (I and II)	105 (40.4)	58 (43.9)	0.667
	High (III)	127 (48.8)	63 (47.7)	
	Unknown	28 (10.8)	11 (8.3)	
HRc status	Positive	164 (63.1)	95 (72.0)	0.079
	Negative	96 (36.9)	37 (28.0)	
HER2 status	Positive	66 (25.4)	46 (34.8)	0.072
	Negative	193 (74.2)	86 (65.2)	
	Unknown	1 (0.4)	0 (0.0)	
Subtype	HRc+/HER2-	131 (50.4)	69 (52.3)	0.137
	HRc+/HER2+	33 (12.7)	26 (19.7)	
	HRc−/HER2+	34 (13.1)	18 (13.6)	
	HRc−/HER2-	61 (23.5)	19 (14.4)	
	Unknown	1 (0.4)	0 (0.0)	
Ki-67	< 10%	133 (51.2)	72 (54.5)	0.705
	≥ 10%	121 (46.5)	56 (42.4)	
	Unknown	6 (2.3)	4 (3.0)	
Type of NST	Anthracyclines	8 (3.1)	6 (4.5)	0.879
	Anthracyclines and Taxanes	239 (91.9)	120 (90.9)	
	Taxanes	10 (3.8)	5 (3.8)	
	Others	3 (1.2)	1 (0.8)	
Anti-HER2 Therapy	Neoadjuvant	12 (4.6)	6 (4.5)	0.968
	Adjuvant	52 (19.9)	25 (18.9)	
	No	196 (75.5)	101 (76.5)	

*AJCC* American Joint Committee on Cancer, *HRc* hormone receptor, *HER2* human epidermal growth factor receptor 2, *NST* neoadjuvant systemic treatment

^a^All sentinel lymph node procedures were performed after neoadjuvant systemic treatment

**Fig. 1:**
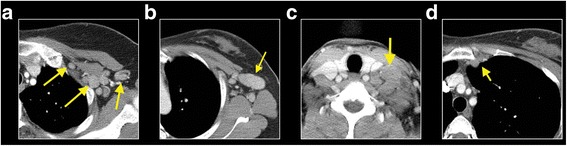
The representative CT images of the regional lymph node enlargements.*Yellow arrows* indicate the presence of the enlarged lymph nodes in axillary level I-III (**a**), a lymph node larger than 2cm in level I (**b**), enlarged supraclavicular lymph node (**c**), and an internal mammary node (**d**)

The nodal status of each patient was assessed using chest CT images obtained before the initiation of the systemic chemotherapy. Among the 392 patients, 69 patients (17.6%) showed no enlarged lymph nodes in the axillary, supraclavicular, or internal mammary nodal chains. We evaluated the lymph node status based on size thresholds (1 cm or 2 cm) and locations (axillary levels I, II, or III, and SCN, or IMN) of the enlarged nodes (Fig. 1).

To estimate the possibility of axillary lymph node involvement in patients who had no visible lymph node enlargement on CT, we analyzed the incidence of lymph node metastasis in 605 early breast cancer patients who underwent chest CT before primary surgery between January 2014 and December 2014 (supplementary cohort A). In this supplementary cohort, all patients initially underwent sentinel lymph node biopsy and only proceeded to axillary lymph node dissection when intraoperative frozen section biopsy identified lymph node involvement. The incidence of lymph node involvement was 17.7%, and most patients had N1 stage disease (Additional file 1: Table S1).

### Development and validation of the CT-based nodal staging system

**Fig. 2:**
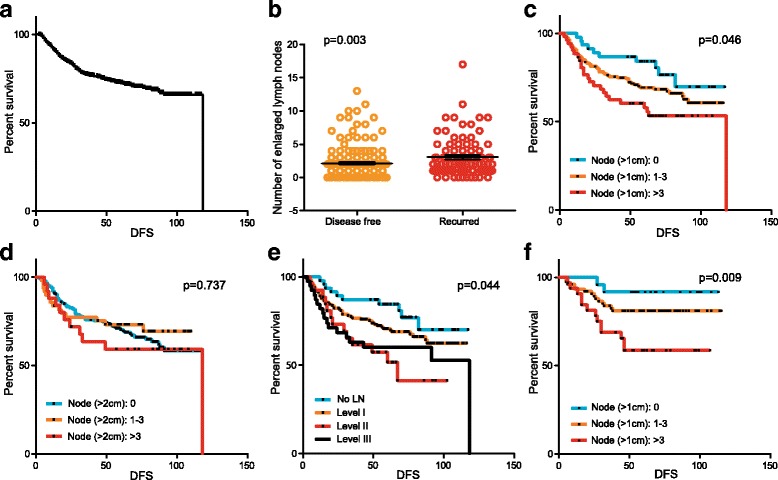
The survival outcome according to the nodal status. The overall disease-free survival in the development cohort of 262 patents (**a**). The number of CT-based enlarged lymph nodes according to the recurrence status (**b**). Various nodal enlargement classification method including 1cm-diameter (**c**), 2 cm-diameter (**d**), and node locations (**e**) are shown. The result of independent validation is shown in **f**

Patients were randomly assigned to the development or validation cohort. The incidence of known prognostic factors did not differ between the development and validation cohorts (Table 1). In the development cohort of 260 patients, we first analyzed the factors associated with tumor recurrence. In this cohort, 88 patients experienced tumor recurrence during the follow-up period (Fig. 2a). As expected, the patients who experienced tumor recurrence had a significantly higher number of enlarged lymph nodes on the pretreatment CT (Fig. 2b). Cox regression analysis showed that an increase in the number of enlarged lymph nodes of more than 1 cm was associated with a 7.2% increased risk of recurrence (Table 2). After observing the prognostic significance, we compared various methods of nodal staging in predicting DFS. Patients were classified according to the number and location of the enlarged nodes. Among the various nodal classification methods, the accuracy of predicting recurrence was highest when the patients were classified according to the number of enlarged lymph nodes (>1-cm diameter, Fig. 2c–e). Also, when comparing with clinical N staging by conventional methods, the concordance rate was very low (kappa value 0.086; 95% CI 0.002–0.170; Additional file 1: Table S3), and the prognostic value of conventional methods was also inadequate (Additional file 1: Figure S1).

**Table 2:** Univariate and multivariate analysis of prognostic factors in the development cohort (*n* = 260)

	Univariate				Multivariate			
	*P*	HR	95% CI for HR		*P*	HR	95% CI for HR	
Age	0.643	0.995	0.973	1.017				
Tumor size	0.027	1.094	1.010	1.184	0.176	1.059	0.975	1.151
Axillary LN (1 cm)	0.006	1.097	1.028	1.172	0.051	1.072	1.000	1.149
High HG	0.001	2.226	1.377	3.598	0.014	1.934	1.146	3.266
HRc negative	0.005	1.841	1.204	2.815				
HER2 positive	0.608	1.132	0.706	1.814				
Subtype^a^	0.072				0.354			
HRc+/HER2+	0.493	0.765	0.357	1.642	0.228	0.617	0.282	1.352
HRc−/HER2+	0.043	1.857	1.050	3.381	0.226	1.469	0.788	2.737
HRc−/HER2-	0.036	1.717	1.035	2.847	0.468	1.222	0.711	2.103
Ki67 ≥ 10%	0.322	1.240	0.810	1.898				

*P* values are derived from univariate or multivariate Cox proportional hazard models

*HR* hazard ratio; *CI* confidence interval; *LN* lymph node; *HG* histologic grade; *HRc* hormone receptor; *HER2* human epidermal growth factor receptor 2

^a^HRc+/HER2- cases were used as reference group

We analyzed the prognostic importance of the CT-based nodal staging system in an independent validation cohort of 132 patients who were treated with neoadjuvant systemic therapy during the same period. In the validation cohort, 29 breast cancer recurrence events occurred during the follow-up period. The staging system could effectively predict the survival outcome when patients were classified according to the number of enlarged nodes >1 cm in size (Fig. 2f).

### Prognostic significance of extra-axillary lymph node enlargement

Traditionally, patients with lymph node metastases in the extra-axillary area are expected to have worse outcomes compared to patients whose lymph node metastases are contained in the axillary area. We assessed the relationship between extra-axillary lymph node enlargement and survival outcome in the entire cohort of 392 patients.

**Fig. 3:**
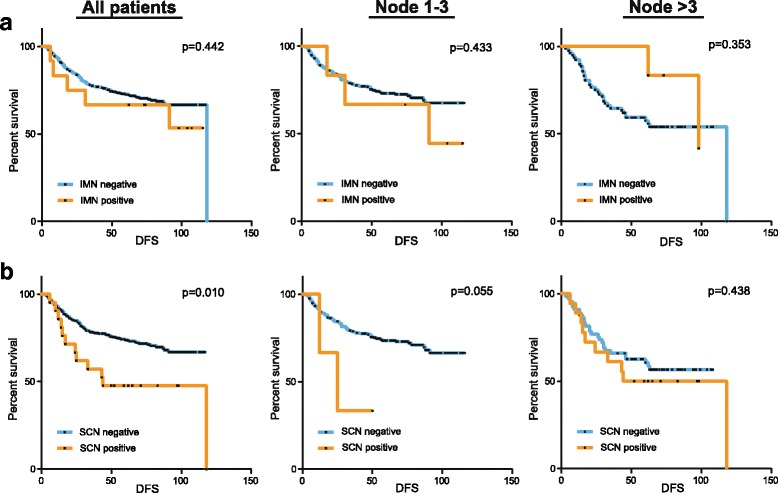
The prognostic importance of the extra-axillary lymph node enlargement. The disease-free survival according to the internal mammary node (IMN) enlargement and supraclavicular node (SCN) enlargement are shown in the Figure **a** and **b**, respectively. Patients were also stratified by the degree of axillary nodal enlargement

Twenty-one (5.4%) and twelve (3.1%) patients had enlarged supraclavicular lymph nodes and internal mammary lymph nodes, respectively. The presence of internal mammary node enlargement was not associated with an increased risk of recurrence regardless of axillary nodal involvement (Fig. 3a). Patients with enlarged supraclavicular lymph nodes showed significantly shorter DFS, but the prognostic significance was lost in patients with more than three enlarged axillary lymph nodes (Fig. 3b). Eighteen patients (85.7%) with enlarged supraclavicular lymph nodes also had more than three enlarged lymph nodes. Our data suggest that the prognostic significance of enlarged supraclavicular lymph nodes was mostly derived from the accompanied axillary nodal involvement.

### Nodal involvement and hormone receptor status

**Fig. 4:**
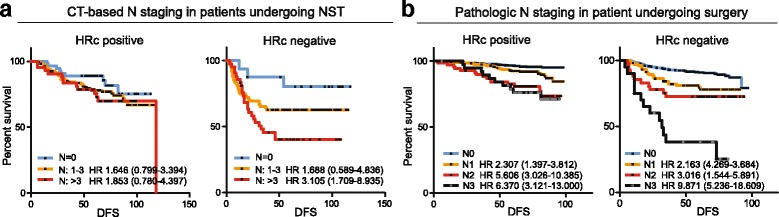
Prognostic significance of the CT-based nodal staging system and the pathologic N stages according to the hormonal receptor status. The prognostic significance of the CT-based nodal staging system in patients undergoing neoadjuvant systemic therapy (**a**) and the significance of the pathologic N stages in patients undergoing primary surgery (**b**)

The prognostic significance of the CT-based nodal staging system was examined in both HRc-positive and HRc-negative patients in the entire cohort. The staging system more clearly predicted the treatment outcomes of patients with HRc-negative tumors (Fig. 4a). To assess whether this HRc-dependent prognostic effect was a limitation of the present CT-based staging system or a result of the intrinsic molecular characteristics of the HRc-positive tumors, we analyzed the prognostic significance of the pathologic N stage in 1702 breast cancer patients who underwent primary surgery between July 2005 and June 2008 at our institution (supplementary cohort B). The basic clinicopathologic characteristics of these patients are described in Additional file 1: Table S2. The relationship between the risk of recurrence and the pathologic N stages in these patients showed similar trends according to HRc status (Fig. 4b) suggesting that the HRc-dependent prognostic implications reflect the biologic characteristics of the breast cancer.

## Discussion

In the present study, we developed and validated an objective clinical N staging method using initial chest CT images of patients undergoing NST. When the patients were classified according to the number of axillary lymph nodes larger than 1 cm, we observed a significant and proportional increase in risk of recurrence. Our data suggest that a CT-based objective axillary staging using the number of enlarged nodes can be a useful alternative to conventional axillary staging done by a physical examination.

Many previous studies have attempted to explore the value of imaging studies, including contrast-enhanced breast magnetic resonance imaging, ultrasonography, and positron emission tomography (PET) scan, in determining nodal status in patients with breast cancer [15]. However, most studies have focused on the role of imaging studies in predicting the presence of nodal metastasis rather than evaluating the quantitative burden of nodal disease. Axillary ultrasound is an imaging modality associated with low cost and risk and is also reported to have high sensitivity in excluding or predicting heavy nodal burden [16–18]. However, quantification of nodal disease using ultrasound is difficult and operator dependent, which compromises the objectivity of the imaging test. The practicality of a PET-CT scan as a nodal staging modality has also been demonstrated in many studies recently [19–24]. For patients undergoing NST, Koolen et al. have demonstrated the role of PET-CT scan for quantification of axillary nodal status and evaluation of extra-axillary nodal involvement [19, 21]. A PET-CT scan performs with high sensitivity and specificity, but its lack of a standard cutoff level for standardized uptake values (SUVmax) and its dependency on the SUVmax of the main tumor are obstacles for objective axillary nodal classification [20, 21, 24]. Moreover, considering the cost and availability of this technique, PET-CT may be best reserved as an adjunct for indeterminate lesions [25]. Compared to these imaging modalities, axillary staging done by chest CT imaging has the advantages of a fairly low cost and high availability. Also, the staging system developed in this study enables physicians to quantify axillary nodal status through an objective and reproducible method for women undergoing NST.

It has been reported that extra-axillary lymph node metastasis, such as SCN and IMN, is associated with poor outcome in patients with breast cancer [9, 26–29]. In our study, we could not demonstrate an independent prognostic value of extra-axillary lymph node enlargement. While patients with enlarged SCN showed worse outcomes, they often had more than three enlarged axillary nodes. In patients with more than three enlarged axillary nodes, the presence of SCN did not confer a significant prognostic difference. Our data suggest that the known prognostic importance of extra-axillary nodal involvement can be the consequence of the degree of axillary metastatic burden rather than an independent prognostic factor. Indeed, Olivotto et al. [30] showed similar overall survival between patients with SCN metastasis and stage IIIB tumors, and Chen et al. [31] showed comparable outcomes between patients with SCN metastasis and N3 stage tumors.

The ability of our staging system to classify patients according to their risk of recurrence was more pronounced in HRc-negative tumors. This phenomenon was also seen in the pathologic nodal staging system for 1702 primary breast cancer patients who received surgery as their initial treatment (Supplementary cohort B). This can be explained by the effect of a higher baseline risk of recurrence for HRc-negative tumors compared to that of HRc-positive tumors, despite a similar increase in the relative risk [32, 33]. On the other hand, the prognostic impact of the degree of nodal involvement may differ according to the molecular characteristics of the breast cancer [34]. The relationship between nodal metastatic burden and the risk of recurrence according to the molecular subtypes of breast cancer should be examined further with a larger dataset.

The retrospective nature of this study is a major limitation. Various CT systems were used for evaluation of enlarged lymph nodes, causing minor differences in slice thickness, resolution, and image quality. A prospective validation study with a standardized protocol is needed to strengthen the value of our nodal staging system. Also, our patient cohort was mainly composed of stage III breast cancer patients (71.2%); therefore, applying our results to early breast cancer patients may have some limitations. Other limitations include the lack of pathologic confirmation of extra-axillary node involvement and the lack of subtype-specific analysis due to the limited number of patients.

## Conclusion

We have developed an objective nodal staging system for patients undergoing NST using the number of enlarged nodes on initial CT images. Our staging system can provide objective and reproducible prognostic information that can overcome the limitations of the current clinical staging system, which relies on the subjective findings of physical examination.

## Additional file

Additional file 1: Figure S1. The survival outcome according to conventional clinical N stage in the development cohort. **Table S1.** The incidence of axillary node involvement in patients with no suspicious nodes on CT (supplementary cohort A). **Table S2.** Clinicopathologic characteristics of patients who underwent primary surgery between July 2005 and June 2008 (supplementary cohort B). **Table S3.** Comparison of the CT-based nodal staging system and conventional clinical N staging (development cohort). (DOCX 83 kb)

## Abbreviations

CI: confidence interval
CT: computed tomography
DFS: disease-free survival
HER2: human epidermal growth factor receptor 2
HG: histologic grade
HR: hazard ratio
HRc: hormone receptor
IMN: internal mammary lymph node
LN: lymph node
NST: neoadjuvant systemic therapy
PET: positron emission tomography
SCN: supraclavicular lymph node
SNUH: Seoul National University Hospital
SUVmax: standardized uptake values
