# Supplementary material for: An objective nodal staging system for breast cancer patients undergoing neoadjuvant systemic treatment
Source: BMC Cancer. 2017 May 31;17:389. doi: 10.1186/s12885-017-3380-8 (PMC5452603; doi:10.1186/s12885-017-3380-8)
Supplement: Additional file 1: Figure S1. — The survival outcome according to conventional clinical N stage in the development cohort. Table S1. The incidence of axillary node involvement in patients with no suspicious nodes on CT (supplementary cohort A). Table S2. Clinicopathologic characteristics of patients who underwent primary surgery between July 2005 and June 2008 (supplementary cohort B). Table S3. Comparison of the CT-based nodal staging system and conventional clinical N staging (development cohort). (DOCX 83 kb) [file 12885_2017_3380_MOESM1_ESM.docx]

Figure S1. The survival outcome according to conventional clinical N stage in the development cohort


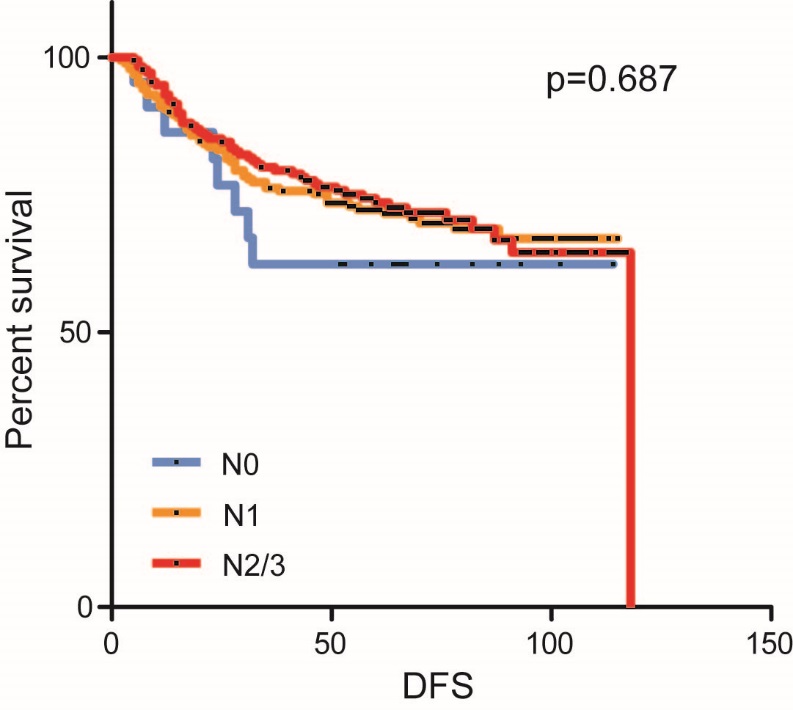


Table S1. The incidence of axillary node involvement in patients with no suspicious nodes on CT (Supplementary cohort A)

| Pathologic N stage |  | n (%) |
| --- | --- | --- |
| N0 |  | 498 (82.3) |
| N1 |  | 93 (15.4) |
|  | 1+ | 68 (11.2) |
|  | 2+ | 18 (2.9) |
|  | 3+ | 7 (1.2) |
| N2 |  | 11 (1.8) |
| N3 |  | 3 (0.5) |
|  |  | 605 |

Table S2. Clinicopathologic characteristics of patients who underwent primary surgery between July 2005 and June 2008 (Supplementary cohort B)

|  |  | Number of patients (%) |
| --- | --- | --- |
|  |  | (n=1,702) |
| Age (median, range) |  | 48 (22 – 89) |
| T stage | T1 | 952 (55.9) |
|  | T2 | 706 (41.5) |
|  | T3 | 44 (2.6) |
|  | T4 | 0 (0.0) |
| N stage | N0 | 1110 (65.2) |
|  | N1 | 419 (24.6) |
|  | N2 | 110 (6.5) |
|  | N3 | 59 (3.5) |
|  | Nx | 4 (0.2) |
| AJCC Stage | I | 751 (44.1) |
|  | II | 765 (44.9) |
|  | III | 186 (11.0) |
| Histology | Ductal | 1529 (89.8) |
|  | Lobular | 35 (2.1) |
|  | Mixed/other | 138 (8.1) |
| Grade | Low (I and II) | 817 (48.0) |
|  | High (III) | 749 (44.0) |
|  | Unknown | 136 (8.0) |
| HRc status | Positive | 1175 (69.0) |
|  | Negative | 527 (31.0) |
| HER2 status | Positive | 269 (15.8) |
|  | Negative | 1346 (79.1) |
|  | Unknown | 87 (5.1) |
| Subtype | HRc+/HER2- | 1018 (59.8) |
|  | HRc+/HER2+ | 96 (5.7) |
|  | HRc-/HER2+ | 176 (10.3) |
|  | HRc-/HER2- | 328 (19.3) |
|  | Unknown | 84 (4.9) |
| Ki-67 | < 10% | 1347 (79.1) |
|  | ≥ 10% | 355 (20.9) |
| Chemotherapy | Yes | 1269 (74.6) |
|  | No | 433 (25.4) |
| Anti-HER2 Therapy | Yes | 91 (5.3) |
|  | No | 1611 (94.7) |
| Radiation Therapy | Yes | 1146 (67.3) |
|  | No | 556 (32.7) |
| Hormone Therapy | Yes | 1168 (68.6) |
|  | No | 534 (31.4) |
| Recurrence | Locoregional | 59 (3.5) |
|  | Distant | 134 (7.9) |
|  | None | 1533 (90.1) |

AJCC, American Joint Committee on Cancer; HRc, hormone receptor; HER2, human epidermal growth factor receptor-2

Table S3. Comparison of the CT-based nodal staging system and conventional clinical N staging (Development cohort)

|  |  | **CT-based nodal staging system** | | |  |
| --- | --- | --- | --- | --- | --- |
|  |  | Node (>1cm):0 | Node (>1cm):1-3 | Node (>1cm):>3 | Total |
| **Conventional clinical N staging** | N0 | 4 | 21 | 21 | 46 |
|  | N1 | 12 | 80 | 70 | 162 |
|  | N2/3 | 1 | 19 | 32 | 52 |
|  | Total | 17 | 120 | 123 | 260 |
